# Supplementary material for: Metabolomic Biomarkers in Bovine Embryo Culture Media and Their Relationship with the Developmental Potential of In Vitro-Produced Embryos
Source: Int J Mol Sci. 2025 Mar 6;26(5):2362. doi: 10.3390/ijms26052362 (PMC11900063; doi:10.3390/ijms26052362)
Supplement: Supplementary file 1 [file ijms-26-02362-s001.zip › Supplemental Table S2.pdf]

Supplemental Table S2. The rates of cleavage, blastocyst formation, and hatching observed in our laboratory.

In vitro development of individually cultured bovine embryos. Cleavage percentages were calculated based on the number of cultured oocytes. Subsequently, blastocyst percentages were derived from the number of cleaved zygotes, and blastocyst expansion and hatching percentages were determined from the total number of blastocysts.

No. of oocytes cultured	No. (%) of oocytes cleaved	No. (%) of blastocysts	No. (%) of expanded blastocysts	No. (%) of hatched blastocysts
600	330 (55)	82 (25)	42 (51)	27 (33)
